# Supplementary material for: Gene Conversion Explains Elevated Diversity in the Immunity Modulating APL1 Gene of the Malaria Vector Anopheles funestus
Source: Genes (Basel). 2022 Jun 20;13(6):1102. doi: 10.3390/genes13061102 (PMC9222773; doi:10.3390/genes13061102)
Supplement: Supplementary file 1 [file genes-13-01102-s001.zip › Figure S2.pdf]

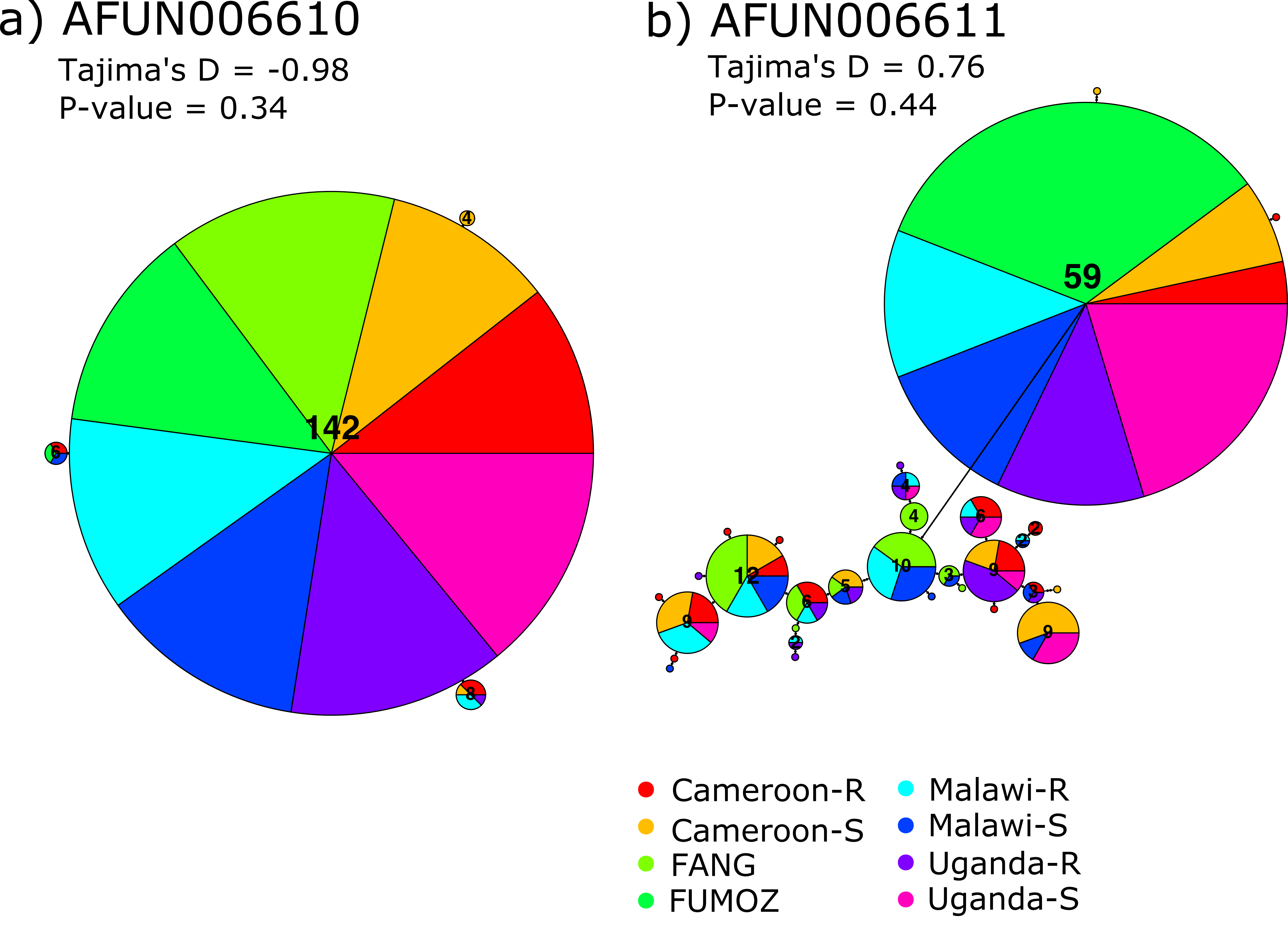
a) AFUN006610
Tajima's D = -0.98
P-value = 0.34
142
4
6
8
b) AFUN006611
Tajima's D = 0.76
P-value = 0.44
59
4
4
10
12
9
6
5
2
3
6
9
2
2
3
9
Cameroon-R
Cameroon-S
FANG
FUMOZ
Malawi-R
Malawi-S
Uganda-R
Uganda-S
