# Supplementary figures and images for: Gene Conversion Explains Elevated Diversity in the Immunity Modulating APL1 Gene of the Malaria Vector Anopheles funestus
Source: Genes (Basel). 2022 Jun 20;13(6):1102. doi: 10.3390/genes13061102 (PMC9222773; doi:10.3390/genes13061102)

# APL1/AFUN018743 Benin

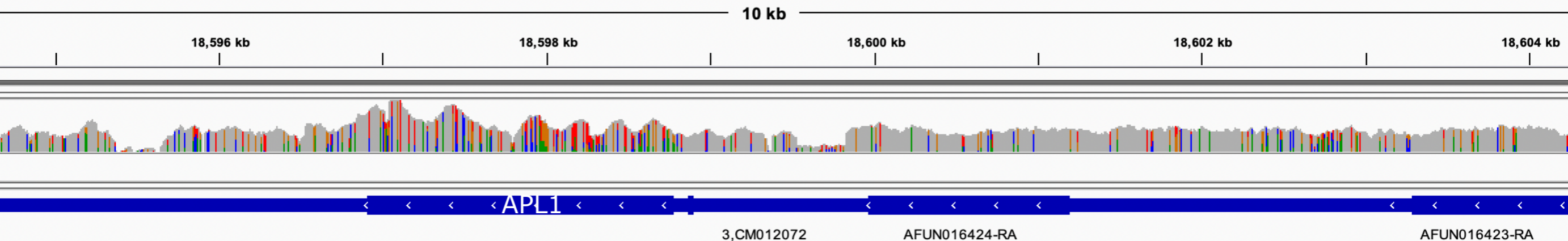

# CYP6AA1 - CYP6AA2 duplication Benin

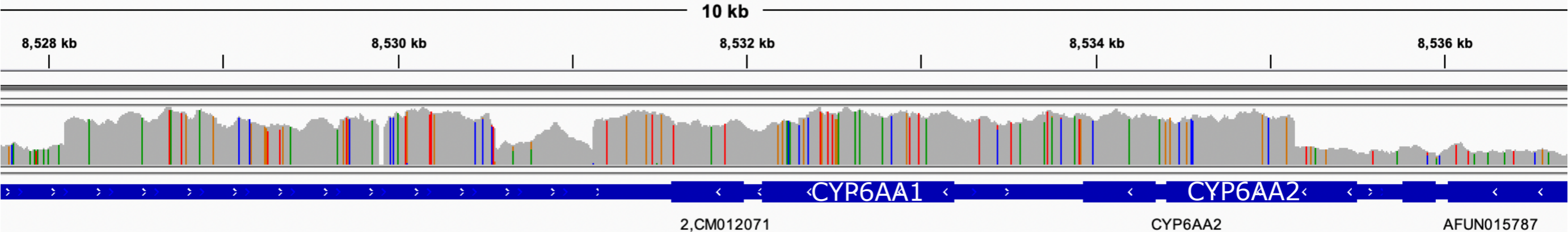

Supplement: Supplementary file 1 [file genes-13-01102-s001.zip › Figure S1.pdf]

AFUN018581

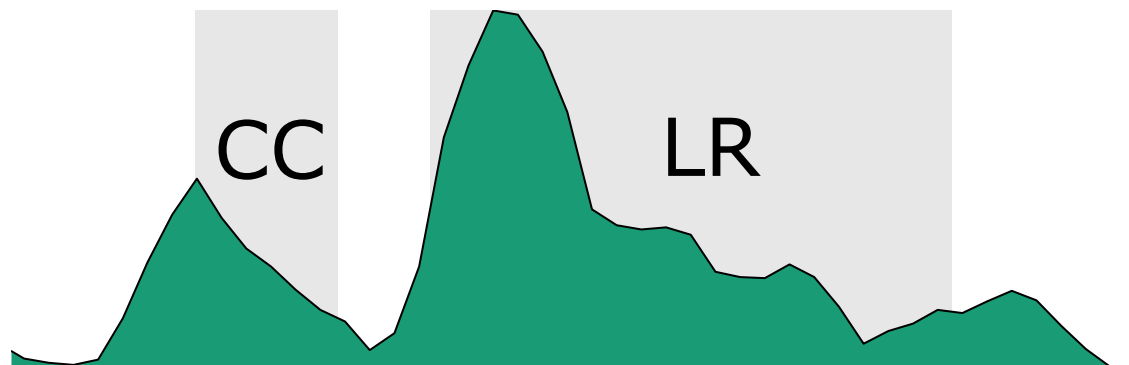

AFUN000288

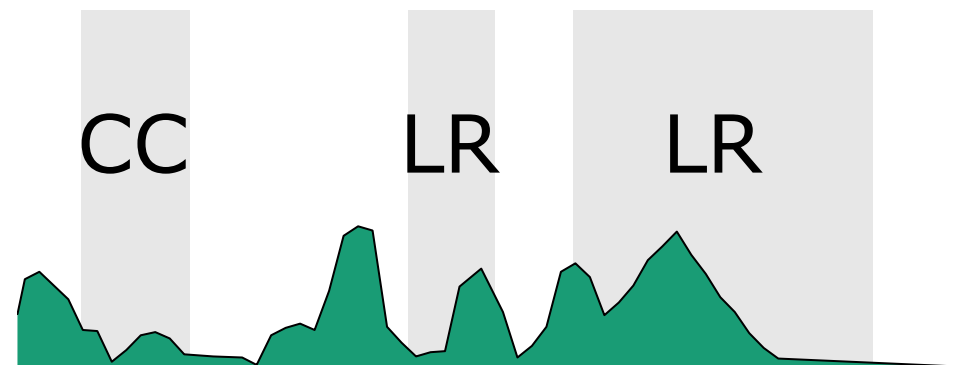

AFUN000279

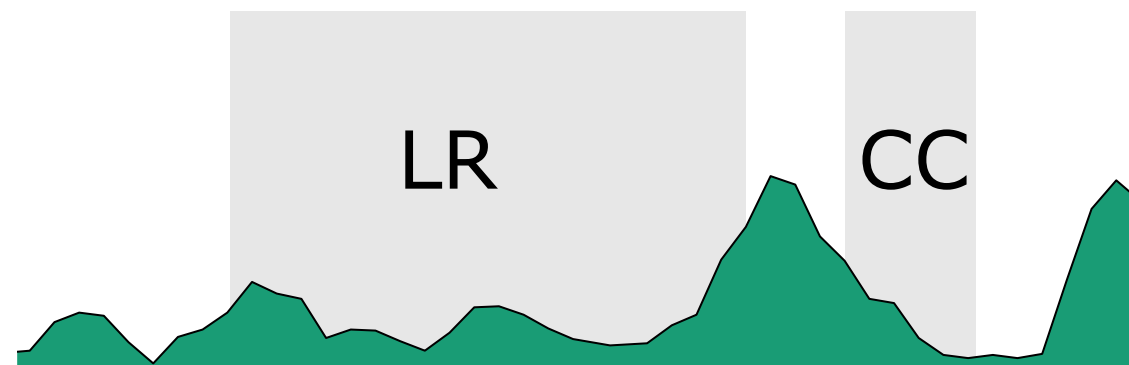

AFUN000597

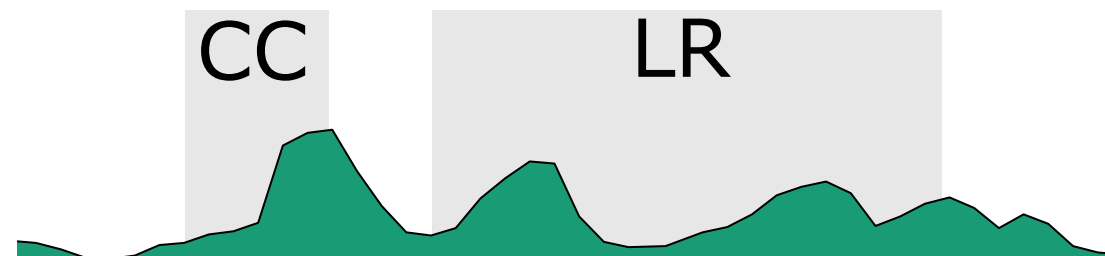

Supplement: Supplementary file 1 [file genes-13-01102-s001.zip › Figure S3.pdf]

# APL1 - AFUN018743

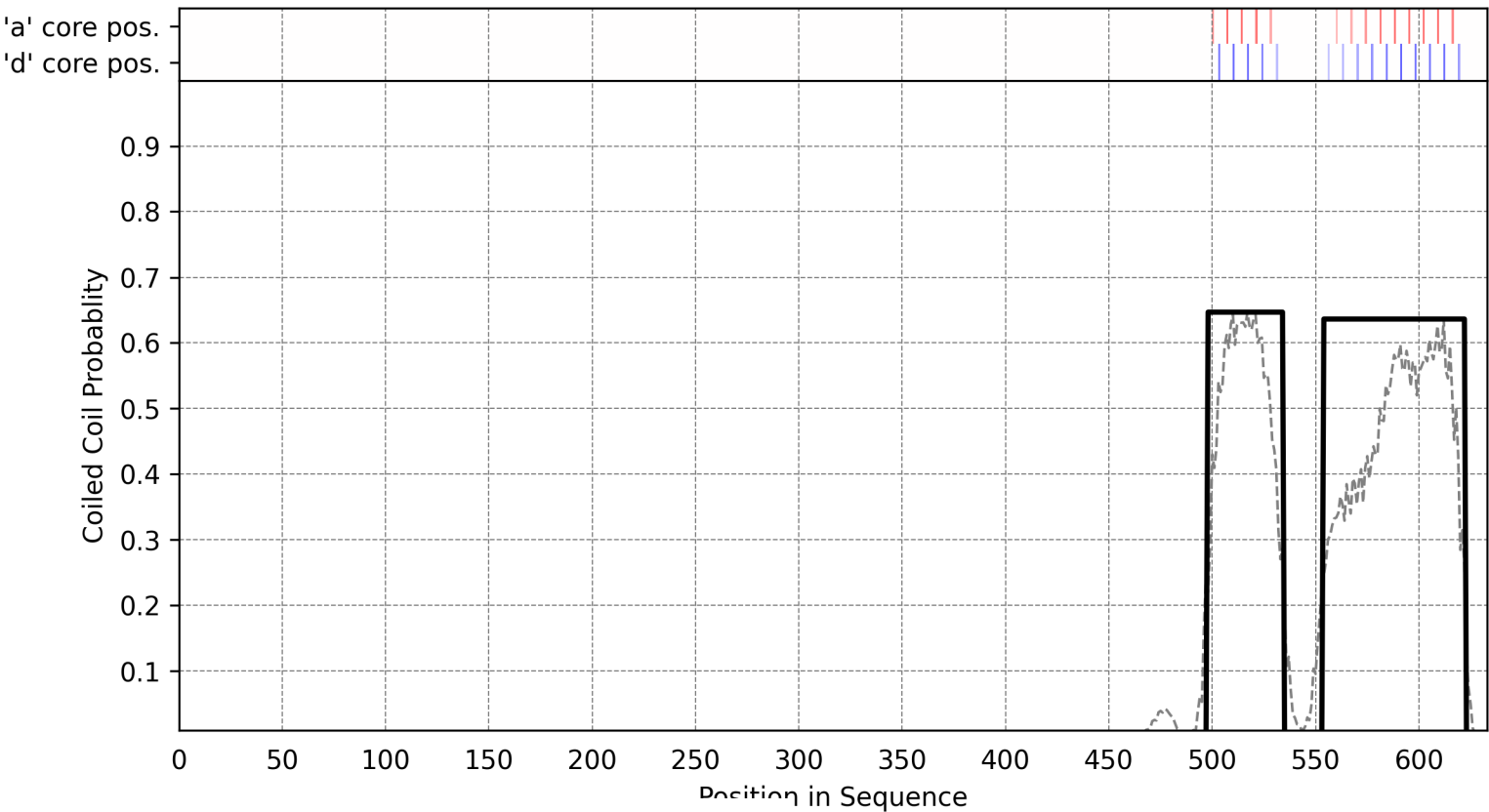

# AFUN000288

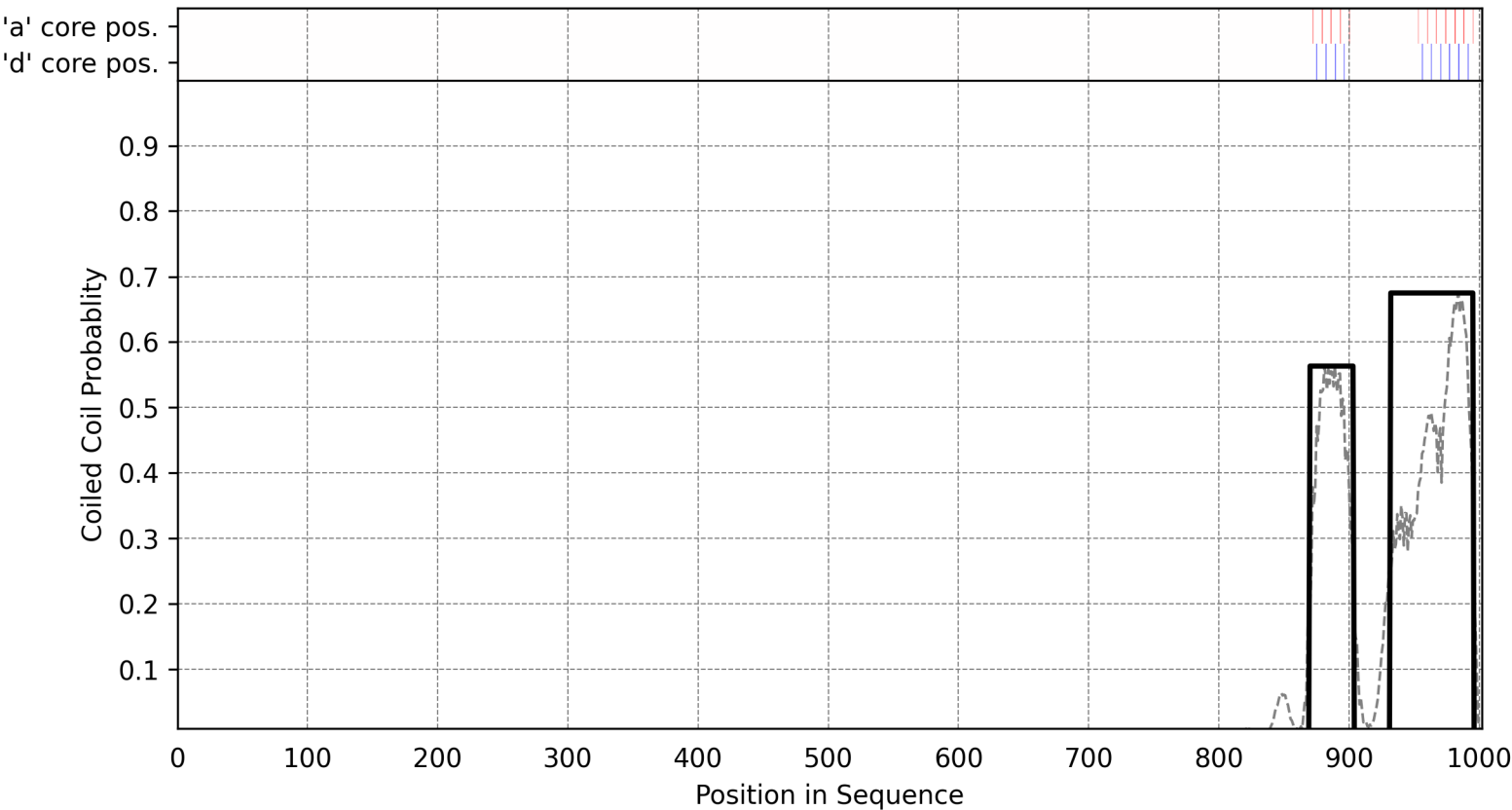

# AFUN000279

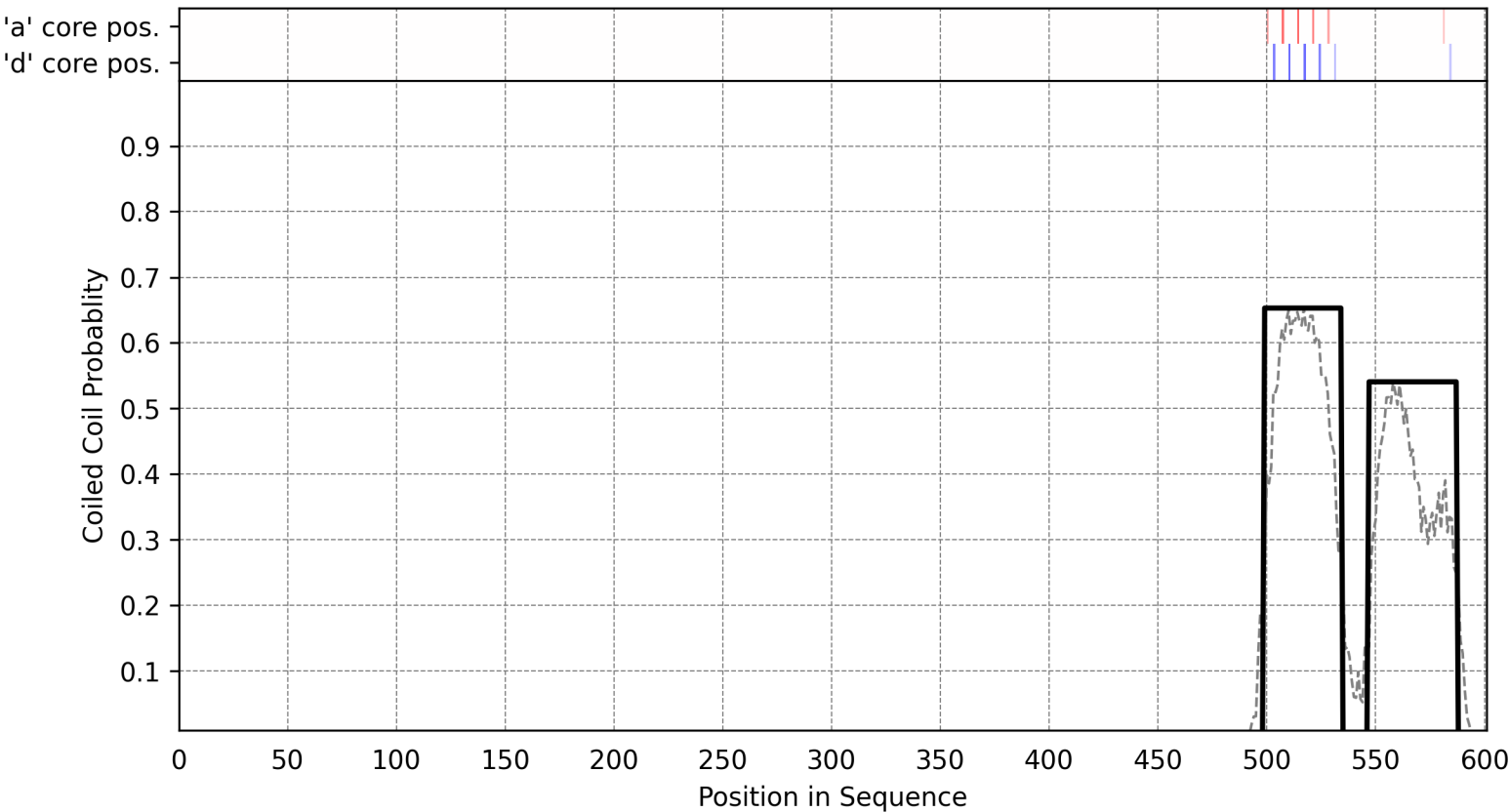

# AFUN000597

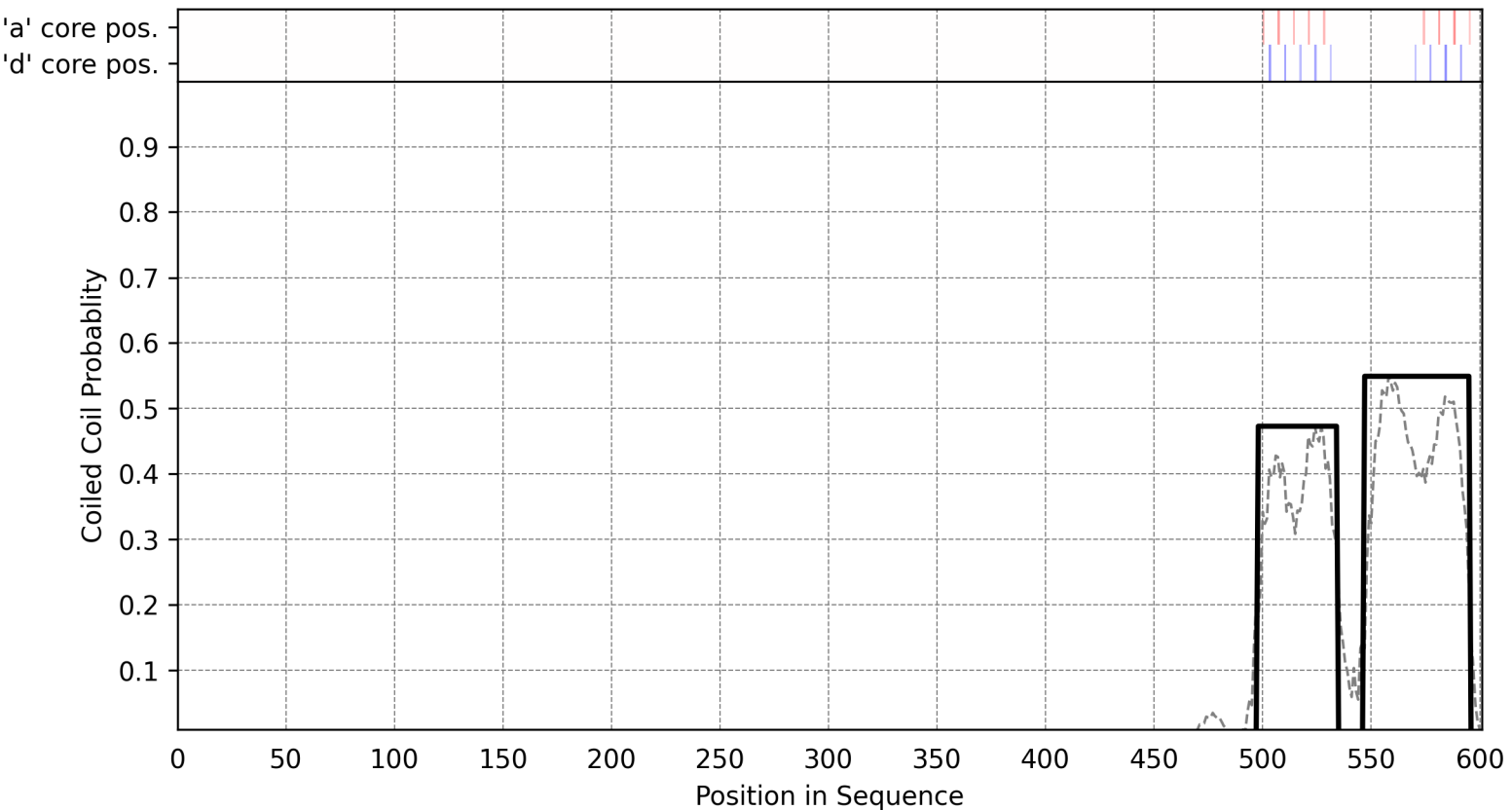

# AFUN018581

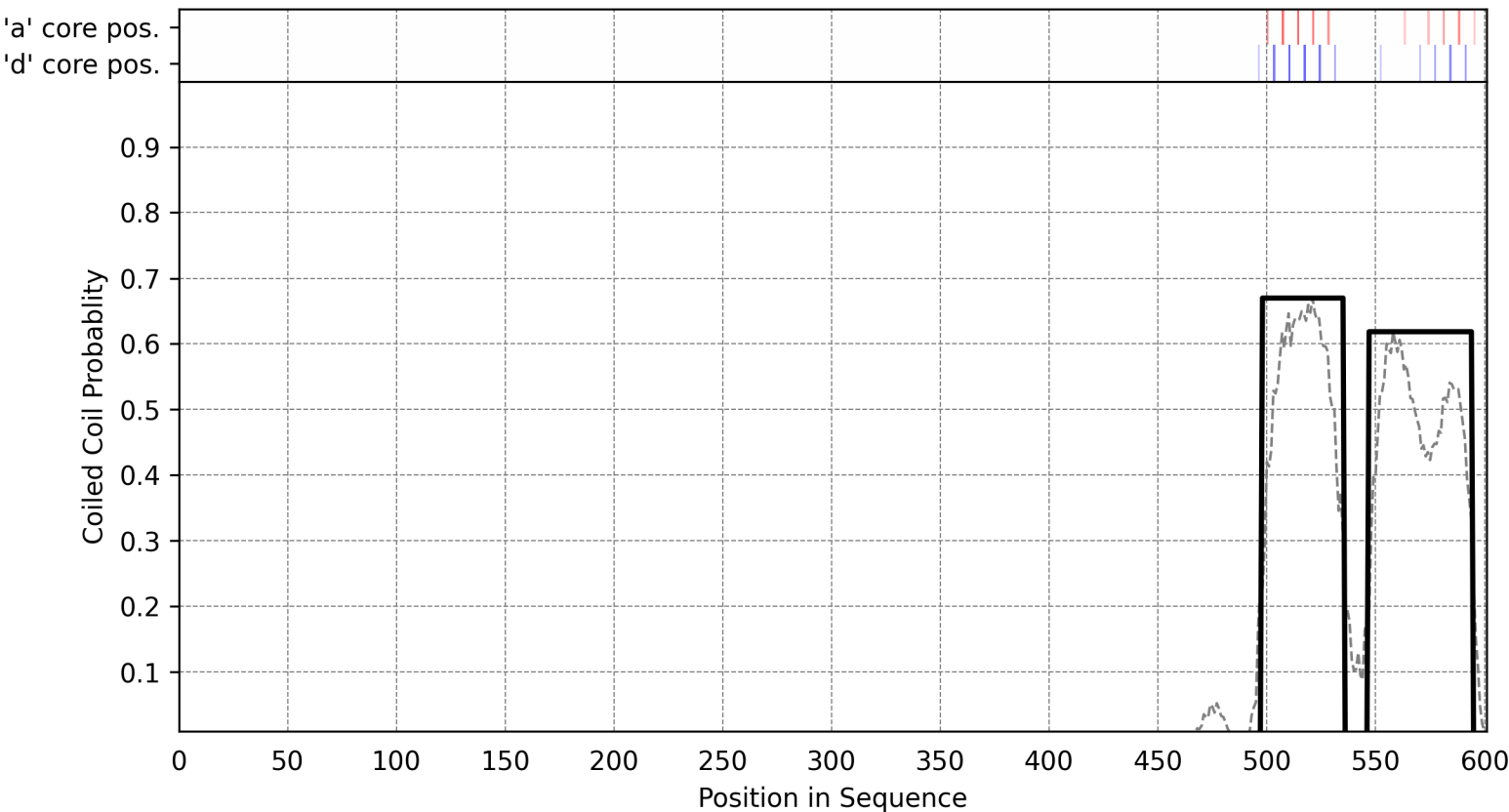

Supplement: Supplementary file 1 [file genes-13-01102-s001.zip › Figure S4.pdf]
